# Regional inequalities in child malnutrition in Egypt, Jordan, and Yemen: a Blinder-Oaxaca decomposition analysis

**DOI:** 10.1186/s13561-016-0097-3

**Published:** 2016-06-07

**Authors:** Mesbah Fathy Sharaf, Ahmed Shoukry Rashad

**Affiliations:** 1 Department of Economics, Faculty of Arts, University of Alberta, Edmonton, Canada; 2 Frankfurt School of Finance and Management, Frankfurt Main, Germany

**Keywords:** Child malnutrition, Rural-urban inequality, Blinder-Oaxaca decomposition, Egypt, Jordan, Yemen, I14, J13

## Abstract

There is substantial evidence that on average, urban children have better health outcomes than rural children. This paper investigates the underlying factors that account for the regional disparities in child malnutrition in three Arab countries, namely; Egypt, Jordan, and Yemen. We use data on a nationally representative sample from the most recent rounds of the Demographic and Health Survey. A Blinder-Oaxaca decomposition analysis is conducted to decompose the rural-urban differences in child nutrition outcomes into two components; one that is explained by regional differences in the level of the determinants (covariate effects), and another component that is explained by differences in the effect of the determinants on the child nutritional status (coefficient effects). Results show that the under-five stunting rates are 20 % in Egypt, 46.5 % in Yemen, and 7.7 % in Jordan. The rural- urban gap in child malnutrition was minor in the case of Egypt (2.3 %) and Jordan (1.5 %), while the regional gap was significant in the case of Yemen (17.7 %). Results of the Blinder-Oaxaca decomposition show that the covariate effect is dominant in the case of Yemen while the coefficients effect dominates in the case of Jordan. Income inequality between urban and rural households explains most of the malnutrition gap. Results were robust to the different decomposition weighting schemes. By identifying the underlying factors behind the rural- urban health disparities, the findings of this paper help in designing effective intervention measures aimed at reducing regional inequalities and improving population health outcomes.

## Background

Chronic malnutrition in early childhood, including the pregnancy period, impairs brain development and has inescapable effects on child growth, such as having an inadequate height for age (stunting) for the rest of their life. Child stunting arises from a combination of poor food consumption, unhealthy environment, and poor health care.^1^ The United Nations Children’s Fund (UNICEF) reported an association between low school performance and nutritional status, which in turn lower employment opportunities and income generation [24].

There is substantial evidence that on average, urban regions have better health outcomes than rural regions in developing countries [25]. Understanding the nature and the underlying factors behind the urban-rural health disparities would help in designing effective intervention measures to improve population health outcomes.

Egypt has the largest number of children under five years old who are stunted in the Middle East and North Africa, and the twelfth worldwide. According to 2008 Egypt Demographic and Health Survey (EDHS), child stunting rate reached 29 %, with 14 % are severely stunted. The 2012 and 2013 Jordan and Yemen Demographic and Health Surveys show that the under-five years stunting rate is 7.7 % in Jordan and 46.5 % in Yemen, the second highest rate in the world. Studies reveal that there are significant disparities in child health outcomes between urban and rural regions in the three countries [12, 21].

The main objective of this paper is to analyze the sources of the malnutrition gap between urban and rural children, in the three countries. A Blinder-Oaxaca Decomposition analysis is used to decompose this gap into two components; one that is explained by differences in the level of the determinants, and another component that is explained by differences in the effect of the determinant on the child nutritional status. The current study aims at informing policy measures in the three countries that combat child malnutrition in rural regions.

The Oaxaca decomposition is a technique that decomposes inequities between any two groups and has been used extensively in explaining wage differentials between males and females, immigrants and natives, blacks and whites workers. The intuition behind the Oaxaca decomposition is that it quantifies the gap in the outcome between the two groups into two parts, a part that is explained by the gap in the level of the determinants, such as income or education level, and a part that is explained by the gap in the effect of the determinants on the outcome variable. For instance, rural children could be less healthy not only because they visit health care providers less frequently but also because health care providers at rural region are less effective. The Oaxaca decomposition quantifies the contribution of each factor to the gap in the outcome, thus identifying which factors contribute most to generating inequality between the two groups [18]. To the best of our knowledge, the current study is the first to examine the disparity in child malnutrition using Oaxaca decomposition in Egypt, Jordan, and Yemen.

The paper is organized as follows: Literature review presents a brief review of the related empirical literature. Methods provides a description of the data and the empirical methodology. Results discusses the results, and Discussion concludes the paper.

## Literature review

It is well documented that children malnutrition is particularly high in developing countries, especially among the least wealthy households, and rural area residents. There is substantial empirical evidence that, on average, rural children have poor health outcomes compared to their urban counterparts [1, 9, 22, 25]. For example, Van de Poel et al. [25] examined children’s nutritional status in 47 countries and found a significant difference between urban and rural children in 43 of the examined countries. Garrett and Ruel [8] and Smith et al. [22] examined whether the socio-economic determinants of children nutritional status differ between urban and rural regions and found that such difference arises from the difference in the nature of characteristics that shapes urban and rural living. They found that rural regions are characterized by more dependence on agriculture activities, less involvement of women in outdoors income generation activities, less female-headed household, and larger household size. Furthermore, rural regions are characterized by lower access to electricity, sanitation and health care. Accordingly, the determinants of children malnutrition could differ due to this difference in the living environment. However, they found no support for the difference in the main socio-economic determinants of child nutritional status between urban and rural regions. They concluded that health disparities between urban-rural children exist because of the gap in the level of the key determinants, as urban regions have more favorable living environment.

Using data from Demographic and Health Surveys (DHS) of 15 sub-Saharan African countries, Fotso [6] found substantial though declining over time, urban–rural differentials in child malnutrition rates. The urban–rural gaps in child malnutrition disappeared once the socio-economic status of the households and communities are controlled for. In another cross-country study of 36 countries from South Asia, Sub-Saharan Africa, and Latin America and the Caribbean, Smith et al. [22] investigated whether the socio-economic determinants of child malnutrition differ between urban and rural regions. They found little evidence for the urban–rural differences in key socio-economic determinants of child nutritional status, including women’s education, access to safe water and sanitation, and household economic status, or in the strength of their association between urban and rural areas.

Using micro-data from the DHS for 47 developing countries, Van de Poel et al. [25] documented the magnitude of the urban- rural disparities in child nutritional status and under-5 mortality. They then adjusted these disparities for differences in the socio-economic characteristics between urban and rural regions. They found considerable urban-rural differences in the average child health outcomes in the entire sample. Controlling for household wealth and socio-demographic factors reduced the urban-rural risk ratios of stunting and under-5 mortality by 75 % and 84 %, respectively. In a considerable number of the covered countries, the urban poor had higher rates of stunting and mortality than their rural counterparts. Using DHS data from 35 developing countries, Fox and Heaton [7] utilized multilevel regression to examine rural-urban differentials in the nutritional status net of the individual, community and national determinants of health status. They found rural children to have a substantially higher risk of poor nutrition, which was mostly attributable to socioeconomic status, access to medical care, and reproductive norms. Srinivasan et al. [23] utilized quantile regression-based counterfactual decomposition methods, to quantify the contribution of different socio-economic determinants to rural-urban gaps in child nutrition outcomes in Bangladesh and Nepal. They found that the urban-rural disparity in child nutrition outcomes was mainly due to differences in the levels of socio-economic characteristics, such as maternal education, spouse’s education, and the wealth index, access to drinking water and sanitation, while differences in the strength of the association between socio-economic characteristics and child nutrition outcomes accounted for less than a quarter of the rural-urban disparities.

Though extant literature is mostly dominated by cross-country studies covering a wide range of heterogeneous countries, a growing number of country-specific studies have investigated the urban-rural disparities in child malnutrition. For example, using a multi-level logistic regression and micro-data from Thailand, Firestone et al. [4] investigated whether the urban-rural disparities in undernutrition and obesity are due to child socioeconomic conditions, or if aspects of the social and physical environment accounted for these disparities, after adjusting for child characteristics. In an unadjusted model, they found statistically significant urban–rural differentials; with urban communities have a lower risk for stunting and underweight. However, the urban–rural differences in the odds of stunting disappeared after controlling for child characteristics, while differences in the odds of underweight remained. Under-nutrition was associated with household poverty. In a recent country-specific study, Kumar and Kumari [13] examined the pattern of rural–urban gap in childhood malnutrition, identified its underlying factors and quantified their contribution, in India during the period 1992 to 2006. They found a considerable widening gap in childhood malnutrition across most rural–urban regions over the study period. They also found the economic status of the household and parental education as the most significant contributors to the rural–urban gap in childhood malnutrition in India.

Few studies have recently explored the socioeconomic disparities in child health in the Middle East. For example, in a recent cross-country study, Krafft and El-Kogali [12] examined the inequality of opportunity in early childhood development in twelve countries in the Middle East and North Africa along several development dimensions. They then quantified the contribution of a set of socioeconomic circumstances to these inequalities. They found substantial inequality of opportunities in early childhood and geographical differences was a key contributor to these inequalities. In another study, Sharaf and Rashad [21] suggested that while Egypt has managed to improve child health, socioeconomic disparities remain substantial. Additionally, they found that the rapid growth in GDP per capita did not result in lower malnutrition rates in Egypt [19].

While a substantial number of studies have examined the urban-rural differentials in nutritional outcomes in a wide range of countries, mainly low-income countries, there is limited evidence, at the population level, in the Arab countries, namely in Egypt, Jordan, and Yemen. Since countries are likely to be heterogeneous with respect to their socio-economic conditions, and level of development, it is to be expected that the factors associated with child malnutrition could be country or regionally-specific and may differ from one country to another. Understanding the nature and the underlying factors behind the urban-rural health disparities would help in designing effective intervention measures to improve population health outcomes.

## Methods

### Data

This paper uses data from the most recent rounds of the Demographic and Health Survey (DHS) for, Jordan, Yemen, and Egypt conducted in the years 2012, 2013, and 2014 respectively. The DHS is an international survey conducted in 85 countries. The surveys present data that allows monitoring and impact assessment indicators in the areas of population, health, and nutrition in the developing countries. The surveys gather information from 12,997 children in Egypt, 5,672 children from Jordan and 12,348 children from Yemen. The DHS samples are nationally-representative household surveys. They have complex survey design that involves stratification based on the level of urbanization and clustering where villages are the primary sampling unit for rural, and districts/towns are the primary sampling unit for urban areas [3]. All the analyses are population weighted using the sampling weights provided in the DHS surveys, and the survey design has been taken into account in the analysis.

The multivariate analyses include a set of Height for Age Z-score (HAZ) determinants that are widely used in the literature. These include child characteristics such as child age, sex; and parental and household-level factors along with other socioeconomic determinants related to the affordability of purchasing nutrition rich food, and living in healthy environment. In particular, the analyses included child sex, age, age squared, mother’s age and level of education, father’s education, household economic status measured by the wealth index, access to piped water, having an independent toilet facility, delivery in a health facility, and parental healthcare visits.

Previous studies suggested that educated mothers could feed their children better, as they are more skilled and could benefit more effectively from health care providers and health care information, and they are more aware of nutrition rich food and the importance of the hygienic living environment. The lack of satisfactory sanitation and safe water supply expose children to the risk of diseases and infections. Satisfactory sanitation is represented by a dichotomous variable indicating whether the household has a toilet, and safe water supply is measured by dummy variable indicating whether a household has access to piped water.

The wealth status, a key socioeconomic determinant, has been consistently linked to health outcome such as malnutrition, as it reflects the household ability to purchase nutrition rich food, living in a healthy environment, and to access health care services. The economic status is measured by wealth index, which is developed by the DHS team. The wealth index is produced by statistical methodology known as the principal components analysis. The value of wealth index is based on household’s ownership of selected assets such as car and TV. Each asset is assigned a weight based on the principle component analysis. Studies have shown that antenatal care is negatively associated with child malnutrition, as it could treat and protect mothers and babies from iron deficiency, anemia, and other diseases. Also, it helps to identify whether the mother needs special care during delivery. Delivery at a health facility helps prevent harmful health consequences, like infections and pregnancy complications. In addition, it provides the necessary information for adequate food intake for the baby and the mother.

### Methodology: Blinder-Oaxaca decomposition

The outcome variable of interest in this study is the HAZ (stunting) in comparison with the World Health Organization’s reference population. To explain the urban-rural disparities in chronic malnutrition among children, we use the Blinder-Oaxaca decomposition. This technique decomposes the gap in HAZ between urban and rural regions into two parts; a part that is due to difference in the distribution of the determinants of HAZ (covariates effect) between the two regions, and another part that is due to the difference in the effect of these determinants (coefficients effect) between the two regions. For example, if *y*_*i*_, our outcome variable, is affected by a single variable, *x*, and we have two groups, urban and rural, then HAZ for the rural, and urban children are given by Eqs. (1) and (2) respectively.

1 $$ {y}_i^{rural}={\beta}^{rural}{x}_i+{\varepsilon}_i^{rural} $$

2 $$ {y}_i^{urban}={\beta}^{urban}{x}_i+{\varepsilon}_i^{urban} $$

Thus the urban-rural gap in the mean HAZ (*y*^*urban*^ − *y*^*rural*^), is given as in Eq. (3).^2^

3 $$ {y}^{urban}-{y}^{rural}={\beta}^{urban}{x}^{urban}-{\beta}^{rural}{x}^{rural} $$

Where *x*^*urban*^ and *x*^*rural*^ are the explanatory variable at their means for the urban and rural. The overall urban-rural gap could be decomposed into a gap that is attributable to difference in the level of the covariates, X's, and a gap that is attributable to difference in coefficients, *β* ' *s* as in Eqs. (4) and (5):

4 $$ {y}^{urban}-{y}^{rural}=\varDelta x{\beta}^{rural}-\varDelta \beta {x}^{urban} $$

5 $$ {y}^{urban}-{y}^{rural}=\varDelta x{\beta}^{urban}-\varDelta \beta {x}^{rural} $$

Where Δ*x* = *x*^*urban*^ − *x*^*rural*^ and Δ*β* = *β*^*urban*^ − *β*^*rural*^. The decomposition equation could be re-written as in Eq. (6):

6 $$ {y}^{urban}-{y}^{rural}=\varDelta x{\beta}^{rural}+\varDelta \beta {x}^{rural}+\varDelta \beta \varDelta x $$

$$ =E+C+CE $$

Where the overall urban-rural gap in child malnutrition is comprised of the gap in endowment (E), and the gap between coefficients(C), and the interactions (CE). Additionally, *y*^*urban*^ − *y*^*rural*^ = *Δxβ*^*rural*^ − *Δβx*^*urban*^ can be equal to (E+(C + CE)) and *y*^*urban*^ − *y*^*rural*^ = *Δxβ*^*urban*^ − *Δβx*^*rural*^ is equal to ((E + CE) + C).

The Blinder-Oaxaca decomposition could be considered a special case of a more comprehensive decomposition Eq. (7).

7 $$ {y}^{urban}-{y}^{rural}=\varDelta x\left(D{\beta}^{urban}+\left(I-D\right){\beta}^{rural}\right)+\varDelta \beta \left({x}^{urban}\left(I-D\right)+{x}^{rural}D\right) $$

Where D is a matrix of weights, and I is an identity matrix. When x is a scalar, the identity matrix would be equal to one. In such case, if the weight (D) equals zero, Eq. (7) would yield Eq. (4), and if D equals one, then we will get Eq. (5) [18]. However, other economists suggest different weights.

For example Reimers [20] suggests weighting the gap in *x* by using the average mean, ${\beta}^{*}=\frac{1}{2}{\beta}^{urban}+\frac{1}{2}{\beta}^{rural}$, while Cotton [2] suggests weighting the *β* by the relative groups sizes, ${\beta}^{*}=\frac{n_{urban}}{n_{urban}+{n}_{rural}}{\beta}^{urban}+\frac{n_{rural}}{n_{urban}+{n}_{rural}}{\beta}^{rural}$. Furthermore, Neumark [16] suggests using the pooled regression coefficients *β*^*p*^ in weighting the difference in *x*, *y*^*urban*^ − *y*^*rural*^ = *Δxβ*^*p*^ + (*x*^*urban*^(*β*^*urban*^ − *β*^*p*^))  + (*x*^*rural*^(*β*^*p*^ − *β*^*rural*^)).

## Results

**Fig. 1:**
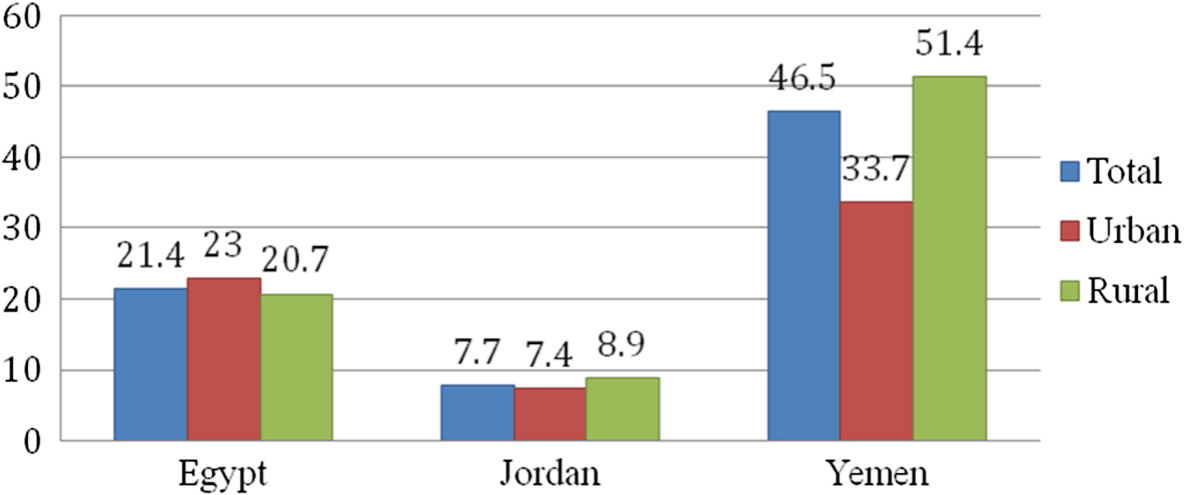
Children Stunting rates in Egypt, Jordan, and Yemen

Figure 1 depicts the aggregate, as well as the urban and rural, child stunting rates in Egypt, Jordan, and Yemen. In Egypt, about one in every five children under five years old is stunted. However, the difference in stunting rates between rural and urban regions is not stark (2.3 %), with rural children have better nutritional status than their urban counterpart. In Yemen, the situation is catastrophic, and the country has substantial high malnutrition rates, with more than half of the Yemen under-5-years children are stunted. Figure 1 shows a significant difference, of about 17 %, in stunting rates between urban and rural children. Jordan is performing much better compared to its neighbors. The overall prevalence of child stunting is quite low (less than 8 %), and the difference in stunting rates between the urban and rural regions is also small (1.5 %) with rural children are performing worse than urban ones.

Table 1 shows the differences in selected background characteristics of households in the urban and rural regions in the three countries. The rural households, are on average, less educated, have lower access to satisfactory sanitation and improved drinking water, and lower access to healthcare than urban households. While the urban-rural differentials are negligible in the case of Jordan, the differentials in the level of the determinants of HAZ between urban and rural regions are apparent in Egypt and are more significant in Yemen. For example, in Yemen, almost half of the urban households in Yemen deliver in a health facility compared to 22.6 % for rural households. The percentage of urban women with secondary or higher education is more than four times the rate of rural women. Substantial difference also exists in the living conditions where 27.2 % of the rural households have improved and non-shared toilet facilities compared to 83.4 % for urban households. Similarly, 49.7 % and 65.2 % of rural households have access to improved water sources and electricity compared to 78.7 % and 98.5 % for urban households respectively. A similar pattern exists in Egypt but with less severity. For example, 59.6 % of the rural women have secondary or higher education compared to 77 % for urban women. 64.4 % and 83.5 % of rural households have access to improved water sources and deliver in a health facility compared to 94.3 % and 93.7 % for urban households respectively.

**Table 1:** Difference in selected background characteristics by urban-rural (%)

Variable	Egypt			Jordan			Yemen		
	Total	Urban	Rural	Total	Urban	Rural	Total	Urban	Rural
Place of delivery: health facility	86.7	93.7	83.5	98.8	98.6	99.3	29.8	49.1	22.6
Women with secondary or higher education	65.7	77	59.6	90.1	91.1	85	12	24.6	6.2
Households using an improved water source	97.8	98.8	97.1	98.9	99.5	96.2	58.8	78.7	49.7
Households with improved. non-shared toilet facilities	76.6	94.3	64.4	99.7	99.8	99.5	44.8	83.4	27.2
Households with electricity	99.8	99.9	99.8	99.5	99.5	99.4	75.6	98.5	65.2

Source: Authors’ compilation based on data from DHS

Table 2 presents the decomposition of the urban-rural malnutrition gap into three components; a gap due to the difference in the level of determinants, a gap due to the difference in the effect of the coefficients and a gap due to the interaction. The first section of the table gives the mean HAZ score in the urban and rural regions, and the HAZ gap between them. Consistent with the results in Figure 1, the HAZ gap in Egypt is insignificant. On the other hand, the difference in HAZ between urban and rural regions in Jordan and Yemen are large and highly significant, particularly in the case of Yemen. Accordingly, the subsequent analysis will focus only on Yemen and Jordan as the HAZ means do not differ significantly between urban and rural regions in Egypt. The second panel of Table 1 divides the HAZ gap into three parts. The endowment part reflects the average increase in rural children HAZ if they had the same level of determinants of the HAZ. The coefficient part shows the change in rural children’s HAZ when using the urban children’s coefficients with the current level of rural children determinants. The interaction component indicates the simultaneous effect of the disparities in the magnitude of the determinants and coefficients [11]. The results show that the gap in child z-score is higher in Jordan than in Yemen. The threefold decomposition analysis in Yemen suggests that the gaps in the means of HAZ are primarily accounted for by the difference in the magnitude of the determinants of child stunting (*p* < 0.001) rather than by differences in their effect. In Jordan, the difference in determinants is important and significant as in Yemen. However, the difference in the effect of coefficient accounts for a large bulk of the z-score gap and is significant at 10 % level of significance.

**Table 2:** Threefold Decomposition

	Yemen	Jordan	Egypt
Differential			
Urban	−143.8***	−35.61***	−57.19***
	[-150.2,-137.3]	[-41.88,-29.34]	[-64.72,-49.66]
Rural	−201.1***	−54.26***	−57.33***
	[-205.1,-197.0]	[-61.97,-46.54]	[-62.23,-52.43]
Difference	57.30***	18.65***	0.140
	[49.71,64.88]	[8.708,28.59]	[-8.848,9.128]
Decomposition			
Endowments	61.47***	7.152*	25.22***
	[52.42,70.51]	[0.517,13.79]	[14.35,36.09]
Coefficients	−25.76**	9.864	−1.628
	[-44.20,-7.315]	[-0.0474,19.78]	[-15.97,12.72]
Interaction	21.58*	1.631	−23.45**
	[2.325,40.84]	[-5.362,8.624]	[-39.46,-7.439]
* N*	12348	5672	12997

95 % confidence intervals in brackets. *p < 0.05, **p < 0.01, ***p < 0.001

Table 3 presents results of the Oaxaca decomposition, using different weights, for both Jordan and Yemen. The first and second columns reflect the decomposition in Eqs. 1 and 2, in which the matrix of weights (D) has a diagonal of weights equals 0, and equals one respectively. The Reimers [20], and Cotton [2] decomposition are in the third and fourth columns respectively. The last column, named *, refers to Neumark [16] decomposition, which uses pooled regression coefficients. The results of Tables 3 suggest that irrespective of the decomposition weight adopted difference in the level of covariates account for the majority of the nutrition gap in Yemen. In addition to the gap in the level of determinants, the difference in the effect of HAZ predictors has a substantial contribution to the malnutrition inequalities in Jordan.

**Table 3:** Decomposition results of the Urban-Rural gap in malnutrition with different weighting schemes

Yemen					
D:	0	1	0.5	0.634	*
Unexplained (U){C+(1-D)CE}:	−4.172	−25.756	−14.964	−10.51	−3.206
Explained (V) {E + D*CE}:	61.469	83.053	72.261	67.807	60.503
% unexplained {U/R}:	−7.3	−45	−26.1	−18.3	−5.6
% explained (V/R):	107.3	145	126.1	118.3	105.6
Jordan					
Unexplained (U){C+(1-D)CE}:	11.495	9.864	10.68	10.154	9.708
Explained (V) {E + D*CE}:	7.152	8.783	7.968	8.493	8.939
% unexplained {U/R}:	61.6	52.9	57.3	54.5	52.1
% explained (V/R):	38.4	47.1	42.7	45.5	47.9

Tables 4 and 5 present the contribution of each determinant in the overall explained rural-urban gap in Yemen and Jordan at different weighting scheme, thus identify which factor explains most of the overall gap. In Yemen, Table 4 shows that, regardless of the adopted weight, income inequality between urban and rural households, measured by the wealth index based on the level of assets ownership, accounts for the majority of the overall gap. The remaining factors have a tiny effect in explaining the HAZ gap in the case of Yemen. For instance, focusing on the Neumark’s decomposition at the last column of Table 4, we find that the gap in the wealth level between urban and rural constitutes 84 % of the explained gap while the remaining correlates explain only 16 % of the overall explained gap. A similar pattern is observed for different weights.

**Table 4:** Which covariates explain most of the urban-rural gap in malnutrition in Yemen?

Variables	E(D = 0)	C	CE	1	0.5	0.294	*
Wealth index	49.172	−10.087	33.222	82.394	65.783	58.927	50.304
Child is twin (yes)	−0.55	0.315	0.224	−0.326	−0.438	−0.484	−0.431
Mother’s malnourished (yes)	1.313	−1.389	0.793	2.107	1.71	1.546	1.553
Pregnancy (yes)	−0.114	−3.123	0.694	0.58	0.233	0.089	0.105
more than one child under five	−0.331	−0.219	0.062	−0.269	−0.3	−0.313	−0.297
Mother’s age (20–24)	0.032	−1.995	−0.016	0.017	0.024	0.028	0.029
Mother’s age (25–29)	0.645	−0.103	−0.011	0.634	0.639	0.641	0.677
Mother’s age (30–34)	0.076	−2.793	−0.029	0.047	0.062	0.068	0.07
Mother’s age (35–39)	−0.231	−2.035	0.093	−0.138	−0.185	−0.204	−0.209
Mother’s age (40–44)	−0.302	0.465	−0.057	−0.359	−0.33	−0.319	−0.331
Mother’s age (45–49)	−0.891	−0.461	0.245	−0.645	−0.768	−0.819	−0.841
Access to clean water (yes)	0.442	4.305	−0.835	−0.393	0.025	0.197	0.179
Child sex (female)	−0.112	1.711	−0.043	−0.155	−0.134	−0.125	−0.128
Child age (one year)	0.498	5.436	−0.144	0.354	0.426	0.455	0.452
Child age (two years)	0.936	13.39	−0.404	0.532	0.734	0.817	0.813
Child age (three years)	−1.039	7.489	0.261	−0.777	−0.908	−0.962	−0.958
Child age (four years)	−2.187	6.607	0.492	−1.695	−1.941	−2.043	−2.036
Mother’s education (primary)	0.449	−0.188	−0.128	0.32	0.385	0.411	0.313
Mother’s education (fundamental)	1.091	3.25	1.599	2.691	1.891	1.561	1.63
Mother’s education (diploma before secondary)	0.183	0.019	0.109	0.291	0.237	0.215	0.201
Mother’s education ( secondary)	1.786	0.208	0.507	2.293	2.039	1.935	1.987
Mother’s education ( diploma after secondary)	0.102	0.076	0.154	0.256	0.179	0.147	0.201
Mother’s education ( university)	3.392	−0.093	−1.077	2.315	2.854	3.076	2.386
Received prenatal care	2.772	1.955	0.599	3.37	3.071	2.948	2.671
Mother’s occupation (professional)	1.632	−0.323	−1.149	0.483	1.057	1.294	0.881
Mother’s occupation (sales)	0.097	−0.098	−0.356	−0.259	−0.081	−0.007	−0.138
Mother’s occupation (agriculture)	0.249	7.367	−7.273	−7.024	−3.387	−1.887	0.193
Mother’s occupation (manual)	−0.018	0.244	0.118	0.1	0.041	0.017	0.014
have a toilet (yes)	0.789	−7.191	−5.602	−4.813	−2.012	−0.856	−0.231
Risky birth interval (yes)	1.59	1.959	−0.457	1.133	1.361	1.455	1.446
_cons	0	−50.465	0	0	0	0	0

**Table 5:** Which covariates explain most of the urban-rural gap in malnutrition in Jordan?

Variables	E(D = 0)	C	CE	1	0.5	0.822	*
Wealth index	9.192	0.427	−0.701	8.49	8.841	8.615	8.927
Child is twin (yes)	−1.294	0.475	0.527	−0.768	−1.031	−0.861	−0.787
Mother’s malnourished (yes)	−0.161	0.244	0.047	−0.113	−0.137	−0.122	−0.119
Pregnancy (yes)	0.098	0.636	−0.082	0.016	0.057	0.03	0.033
More than one child under five	0.208	3.83	−0.616	−0.408	−0.1	−0.299	−0.281
Mother’s age (20–24)	−0.098	4.16	1.305	1.206	0.554	0.974	1.116
Mother’s age (25–29)	−0.009	6.067	0.013	0.003	−0.003	0.001	0.003
Mother’s age (30–34)	0.585	8.766	−1.086	−0.501	0.042	−0.308	−0.414
Mother’s age (35–39)	0.176	4.864	−0.336	−0.16	0.008	−0.1	−0.15
Mother’s age (40–44)	0.076	0.854	0.088	0.164	0.12	0.148	0.19
Mother’s age (45–49)	−0.104	0.658	0.18	0.076	−0.014	0.044	0.06
Access to clean water (yes)	−0.052	−6.069	0.587	0.535	0.241	0.43	0.433
Child sex (female)	−0.028	1.796	0.039	0.011	−0.008	0.004	0.005
Child age (one year)	2.362	4.709	−0.967	1.396	1.879	1.568	1.612
Child age (two years)	−0.378	5.794	0.163	−0.214	−0.296	−0.243	−0.247
Child age (three years)	−2.786	1.893	0.604	−2.182	−2.484	−2.29	−2.329
Child age (four years)	−0.997	7.139	0.51	−0.486	−0.742	−0.577	−0.582
Mother’s education (primary)	−0.164	1.755	−0.429	−0.593	−0.378	−0.516	−0.411
Mother’s education ( secondary)	1.24	12.01	2.223	3.463	2.352	3.068	2.663
Mother’s education ( university)	−1.078	11.057	−1.519	−2.597	−1.837	−2.326	−2.081
Received prenatal care	0.041	30.354	0.612	0.653	0.347	0.544	0.481
Mother’s occupation (professional)	−0.378	−2.691	0.834	0.456	0.039	0.308	0.317
Mother’s occupation (sales)	0.301	0.3	−0.232	0.069	0.185	0.11	0.198
Mother’s occupation (agriculture)	0.023	0.237	−0.023	−0.001	0.011	0.003	0.002
Mother’s occupation (manual)	−0.012	0.148	−0.024	−0.036	−0.024	−0.032	−0.034
Risky birth interval (yes)	0.391	0.937	−0.086	0.304	0.348	0.32	0.333
_cons	0	−90.488	0	0	0	0	0

In Jordan, Table 5 shows that the wealth gap comprises the largest bulk of the gap in endowments regardless of the applied weight. The difference in the coefficients comprises a large part of the nutrition status gap. The effect of the socio-demographic variables (child’s age, mother’s age, and child sex) disfavors the rural children. Interestingly, the difference in the wealth index effect under the coefficient column favoring the rural residents, indicating that rural households use their wealth more efficiently in improving their child health. Table 5 shows that mother’s education has a higher return in urban regions compared to rural regions. This could to the difference in the quality of graduates between urban and rural areas. More importantly, the analysis shows the difference in the effect of receiving prenatal care between urban and rural regions has a considerable contribution to the coefficient gap. This has an important policy implication, as it might be that the quality of health care in the urban areas largely outperforms the rural regions driving this significant difference in child health between the regions. Wealth index has a trivial contribution to the coefficient, which implies rural household are not efficient in using their assets. Thus, the conclusion here is that children in rural areas are less healthy mainly due to the lower return of education and health care service in the rural regions in Jordan, which may reflect some discrimination.

## Discussion

In this study, we comprehensively investigated the underlying proximate, demographic and socioeconomic factors that account for the rural-urban inequalities in child malnutrition in Egypt, Jordan, and Yemen, on which limited research has been conducted, using a Blinder-Oaxaca decomposition analysis.

Data from the DHS show that the overall under-five stunting rate is 20 % in Egypt, 46.5 % in Yemen, and 7.7 % in Jordan. The rural- urban gap in child malnutrition was minor in the case of Egypt (2.3 percentage points) and Jordan (1.5 percentage points) while the regional gap was substantial in the case of Yemen (17.7 percentage points).

We found marked rural-urban disparities, though not uniform, across the three countries, in the level of socioeconomic determinants of children’s nutritional status. In general, rural households, are less educated, have lower access to satisfactory sanitation and improved drinking water, and lower access to healthcare than urban households. The urban-rural differentials in the level of the socioeconomic determinants of HAZ were substantial in Yemen and to a lesser degree in Egypt and are negligible in the case of Jordan.

While the current study has documented considerable differences, on average, between urban and rural regions, it is essential not to ignore the heterogeneity of urban and rural communities when designing intervention measures. Several studies have reported large intra-urban and intra-rural differences in socioeconomic conditions. For example, in an earlier cross-country study, Menon et al. [15] found that for some of the examined countries, intra-rural differences in stunting rates were much greater than intra-urban differences. Urban children from the lowest income quintiles were ten times more likely to be stunted compared to children from the highest income quintiles while the intra-rural differential in stunting risk by income quintiles was much less.

Identifying whether the rural-urban gap in child malnutrition is more depending on differences in the level of the determinants (covariates effects), or on differences in the effects of the determinants (coefficients effects) is crucial for designing the appropriate intervention measures and policies aiming at reducing health inequalities. For example, if the rural-urban health gap is due to differences in the effect of the determinants, then, the redistribution of wealth and improving access to healthcare, clean water and sanitation would not be sufficient to improve child health in rural areas, since the impact of these interventions are weaker in rural areas. Behavioral and awareness programs would be necessary interventions to close the gap between the two groups. However, if the rural-urban health gap is due to differences in the level of the determinants, then redistribution of wealth and improvement in the level of the determinants in rural areas would be an effective policy to reduce regional health inequalities. In an earlier study, Jalan and Ravallion [10] examined the impact of access to piped water on child health in rural India. They suggest that expanding piped water does not necessarily guarantee child health improvement, as the outcome of public investment also depends on the parental behavioral responses. Though public investments have been favoring the poor, children from the low socio-economic status may benefit less from access to medical care or piped water because their parents are less knowledgeable about how to reap the maximum benefits from medical care or piped water. They will have lower coefficients in comparison to parents from high socio-economic status who for example can read child drug instructions and take full advantage of it, or know better how to use piped water.

The merit of using the Blinder-Oaxaca decomposition is that it accounts for group differences in the effects of the determinants (coefficient effects), as well as differences in the effect of the determinants across urban and rural families, which would unmask the source of the regional inequalities and help design effective intervention measures.

In the current study, results of the Blinder-Oaxaca decomposition suggest that the rural-urban gap in child malnutrition in Yemen is primarily explained by the difference in the magnitude of the determinants of child nutritional status (covariate effect is dominant), rather than by differences in their effect. In particular, income inequality between urban and rural households, measured by the wealth index, accounts for most of the overall gap. This is consistent with the study of Larrea and Kawachi [14] on economic inequality and child stunting in Ecuador. They suggested that economic inequality has significant negative impact on child stunting. This implies that in Yemen, redistribution of wealth, and improving access to healthcare, clean water and sanitation in rural areas would be effective to reduce the regional inequalities in child malnutrition and improve child health in rural areas. However, in Jordan, though the difference in the determinants is important and significant as in Yemen, the difference in the effect of determinants accounts for a large bulk of the z-score gap and is significant (coefficient effects dominate). These results are robust to the different decomposition weighting schemes. Accordingly, the redistribution of wealth and improvement in the level of the determinants in rural areas in Jordan may not be sufficient to reduce regional health inequalities. Behavioral and awareness programs that would increase the effect of the determinants would be necessary interventions to close the rural-urban gap in child malnutrition in Jordan.

Since the seminal work of Oaxaca and Blinder in the early 1970s, several decomposition methods, including those going beyond the mean, have been developed to decompose the difference in a distributional statistic between two groups, or its change over time. For a comprehensive review of the various decomposition methods in economics, see Fortin et al. [5]. In general, each decomposition method has its own underlying assumptions, advantages and limitations. One limitation that several decomposition methods face is path dependence, that is, the decomposition results depend on the order in which the decomposition is conducted. In addition, some methods such as, quantile regression methods, are computational intensive and may fail to provide a detailed decomposition for distributional parameters other than the mean.

An alternative approach to the Oaxaca–Blinder approach for analyzing rural-urban differences in the impact of child nutritional determinants would be the use of dummy variable interaction effects in a conventional multiple regression framework. The disadvantage of this approach is that it may not be possible to include as many interactions as there are covariates while still retaining a meaningful interpretation of the coefficients. Several other studies use stepwise regression type approaches, however, this is difficult to use if we are interested in quantifying the relative contributions of all the determinants to the outcome gap. In addition, results of this approach are sensitive to the order in which we control for the covariates, which is not the case in the Oaxaca–Blinder approach.

The Oaxaca–Blinder decomposition technique has several limitations. One limitation is the “index number problem” in which the choice of the reference group could affect the ratio of explained to unexplained portions of the rural-urban gap in child malnutrition. Another problem is the “indicator variable” problem, where results related to dummy variables in the model might be sensitive to which group is the reference or the omitted group. For the unexplained portion of the gap, we may not differentiate the part attributed to the group membership (true “unexplained” captured by the difference in intercepts) from the part attributed to differences in the coefficient of the omitted group [5]. Another limitation of the Oaxaca–Blinder decomposition is that it might be difficult to make inference about the main cause of the unexplained part of the rural-urban gap in child malnutrition.

The Oaxaca–Blinder approach that is used in the current study decomposes the difference in the mean HAZ scores between two groups (rural versus urban children). Group differences in other parameters of the HAZ distribution could also be of interest. One extension to the current study would be to use quantile regression to decompose the regional differences in the full distribution of the HAZ score into the contribution of differences in the distributions of the covariates, and differences in the effects of these covariates. Among the studies that used that approach see for example O’Donnell et al. [17]. The merit of decomposing the full HAZ distribution is that it allows the effect of the covariates to differ over the conditional distribution of the HAZ score which would be more informative for policy guidance.

The current study is not free from limitations. One limitation is its cross-sectional design which limits the ability to make causal inferences and examine how the regional inequalities evolve over time. The rural-urban disparities in health could be evolving over time as a result of urbanization and other areas of socioeconomic development. Another limitation is that there could be other confounding factors, whether observed or unobserved, that could explain the rural- urban differentials and that we did not control for.

One problem that is common in the analysis of rural-urban differentials involves the way localities are classified as urban or rural. There are several urban and rural classification criteria such as administrative boundaries, population size, population density, the percentage of the labor force in non-agricultural activities. The lack of a uniform classification for urban and rural regions could potentially hinder making cross-country comparisons.

## Conclusion

In this study, we decomposed, and identified the underlying factors, behind the rural- urban gap in child malnutrition in Egypt, Jordan, and Yemen. This is to inform and guide policies aimed at reducing health inequalities and improve population health in the Arab world. In Yemen, the regional gap in child malnutrition is mostly due to differences in the level of the determinants of child nutritional status, rather than by differences in their effect. Accordingly, intervention measures that entail redistribution of wealth, and improving access to healthcare, clean water and sanitation in rural areas would be effective to reduce the regional inequalities in child malnutrition. In Jordan, group difference in the effect of the determinants accounts for a significant fraction of the z-score gap. This implies that polices that improve the level of the determinants in rural areas in Jordan may not be sufficient to reduce regional health inequalities. Behavioral and awareness programs that would increase the effect of the determinants would be necessary interventions to close the rural-urban gap in child malnutrition in Jordan.

## Footnotes

1 Child stunting is measured by comparing child length for age with a reference population, where a child whose length for age is less than minus two standard deviation of a reference population is considered stunted.

2 Assuming exogeneity, the conditional expectations of the error terms in Eqs. 1 and 2 are zero.
